# Inflammation-Driven Remodeling of the Blood–Testis Barrier: Roles of Junctional Complexes, Actin Dynamics, and Kinase Signaling

**DOI:** 10.3390/biomedicines14020423

**Published:** 2026-02-13

**Authors:** Zoltán Virág, András Nagy, Viktória Kiss, Denise Börzsei, Csaba Varga, Renáta Szabó

**Affiliations:** Department of Physiology, Anatomy, and Neuroscience, Faculty of Science and Informatics, University of Szeged, H-6726 Szeged, Hungary; zolee0920@gmail.com (Z.V.);

**Keywords:** blood–testis barrier, inflammation, male infertility, cytokines, experimental models

## Abstract

The blood–testis barrier (BTB) is a highly specialized and dynamic junctional structure formed by adjacent Sertoli cells that is essential for maintaining testicular immune privilege and supporting spermatogenesis. While the BTB undergoes tightly regulated, stage-dependent remodeling under physiological conditions, inflammatory stimuli can profoundly disturb this process. Accumulating evidence indicates that inflammatory conditions disrupt BTB integrity by altering junctional protein organization, cytoskeletal dynamics, and barrier permeability. We aimed to integrate current evidence to elucidate the key pathways by which inflammation impairs BTB integrity, drawing on studies using intratesticular administration of pro-inflammatory cytokines and experimental rodent models of reproductive dysfunction characterized by pathological inflammation, including chemotherapy-induced inflammation and orchitis. Collectively, findings from these models demonstrate that inflammatory signaling compromises BTB integrity, destabilizes the spermatogenic niche, and may contribute to impaired spermatogenesis. Our narrative review frames the BTB as a dynamic and inflammation-sensitive structure whose regulation emerges from the coordinated action of inflammatory pathways, cytoskeletal remodeling, and junction-associated signaling modules, rather than from isolated molecular events.

## 1. Introduction

A growing body of evidence indicates that elevated inflammatory signaling in the male reproductive tract plays a pivotal role in testicular dysfunction and reproductive health, both in humans and in experimental models [1,2]. The blood–testis barrier (BTB), formed by adjacent Sertoli cells, is crucial for maintaining testicular function by segregating the seminiferous epithelium into basal and adluminal compartments, thereby establishing an immune-privileged environment essential for spermatogenesis [3,4]. Its structural and functional integrity is tightly regulated by cytoskeletal dynamics and protein kinases, among which Src family kinases (c-Src, Fyn, c-Yes) act as critical modulators of junctional protein turnover, actin organization, and barrier remodeling, integrating extracellular inflammatory signals with intracellular cytoskeletal responses [5,6]. Disruption of BTB integrity is directly associated with spermatogenic failure and male infertility, with altered localization and/or expression of key BTB proteins, including claudin-11, occludin, and connexin-43, contributing to impaired reproductive outcomes [7,8].

This narrative review aims to provide a comprehensive overview of the mechanisms by which inflammatory signaling regulates BTB dynamics, with a particular focus on the interplay among inflammation, BTB proteins, kinase signaling and cytoskeletal interactions. Here, we examined the interplay between inflammation and the BTB through two complementary approaches. First, we focus on rodent models in which exogenously administered cytokines act as direct inflammatory mediators to perturb BTB structure and function. Second, we highlight experimental rodent models of reproductive dysfunction in which pathological inflammation—arising from disease-associated processes—compromises BTB integrity. Together, these two thematic perspectives provide a comprehensive framework to understand how inflammatory signaling influences BTB dynamics and male reproductive health.

To contextualize the scope and limitations of the reviewed evidence, this narrative review was based on a structured literature search of the PubMed and Web of Science databases using combinations of keywords, including “blood–testis barrier”, “inflammation”, “cytokines”, “Sertoli cells”, and “Src family kinases”. Priority was given to peer-reviewed original research articles that provided mechanistic insights into BTB regulation under inflammatory or pathological conditions. While no formal risk-of-bias assessment was applied, studies were selected based on experimental rigor, relevance to BTB biology, and consistency with established concepts of Sertoli cell junctional dynamics. The presented literature is primarily composed of rodent models and studies reporting positive or mechanistically interpretable findings, which may introduce selection and publication bias. Experimental paradigms involving intratesticular cytokine administration or chemotherapeutic injury may not fully reflect the temporal complexity and systemic features of chronic inflammatory conditions observed in humans. These considerations highlight the need for cautious interpretation when extrapolating experimental findings to human male reproductive pathology, while also underscoring the value of rodent models for dissecting conserved molecular mechanisms underlying inflammation-induced BTB remodeling.

## 2. The Structure and Function of the Blood–Testis Barrier

Testicular function is controlled by the hypothalamic–pituitary–gonadal (HPG) axis, which integrates central neuroendocrine signaling with local cellular specialization within the testis. Pulsatile secretion of gonadotropin-releasing hormone (GnRH) from hypothalamic neurosecretory neurons represents the primary driving force of this axis and is essential for maintaining reproductive competence. GnRH induces luteinizing hormone (LH) and follicle-stimulating hormone (FSH) release from the anterior pituitary. LH acts on Leydig cells to promote testosterone synthesis, while FSH supports germ cell development via Sertoli cells (SCs). Beyond their classical endocrine roles, LH- and FSH-mediated signals directly influence SC maturation and junctional remodeling, processes that are essential for the establishment of the highly specialized barrier, the blood–testis barrier (BTB), which creates an immune-privileged microenvironment required for sustained spermatogenesis and germ cell survival [9].

The BTB represents one of the most complex and dynamic cell–cell junctional systems in the mammalian body, formed by adjacent SCs within the seminiferous epithelium. Accumulating evidence indicates that BTB formation is tightly linked to the pubertal increase in gonadotropins, particularly FSH and LH, as the absence or experimental withdrawal of these hormones markedly delays or completely inhibits barrier establishment [10]. This process is believed to coincide with the cessation of SC proliferation and the onset of their functional maturation [11]. In rodents, this transition occurs during the late neonatal to early prepubertal period, with terminal differentiation and functional BTB establishment taking place between postnatal days 17 and 21 [12,13]. The transition from an immature precursor state to a fully differentiated, adult SC typically occurs during puberty [14]. In humans, Sertoli cell maturation follows a more prolonged trajectory, with an additional peri-pubertal proliferative phase and final differentiation occurring at the onset of puberty, generally between 12 and 14 years of age [10,12,15]. Immature fetal SCs can retain the capacity to undergo functional maturation even after transplantation into adult testes, which highlights the remarkable developmental plasticity of SCs and underscores their capacity to establish the structural framework required for barrier formation [16].

First described by Enrico Sertoli in 1865, SCs extend from the basement membrane to the lumen of the seminiferous epithelium, forming two distinct compartments. The basal compartment, located between the basement membrane [17] and the SC tight junctions, contains spermatogonia, as well as preleptotene (early meiotic) spermatocytes prior to their transit across the BTB, thereby supporting early germ cell differentiation while maintaining immune protection. Because germ-cell development requires the spatial segregation of mitotic and meiotic phases, the structure and dynamic regulation of the BTB are intimately linked to the progression of spermatogenesis within the seminiferous epithelium. Spermatogenesis is a continuous, tightly regulated process in which diploid spermatogonia differentiate into haploid spermatozoa within the seminiferous tubules, relying on coordinated interactions with SCs and the BTB [18]. In rats, the spermatogenic cycle comprises fourteen stages (I–XIV), during which the BTB undergoes dynamic remodeling to support germ cell development. In stages I–VI, the BTB remains stable with bundled actin supporting early meiosis and spermatid differentiation. Stage VII marks the onset of junctional and cytoskeletal reorganization, while stage VIII allows preleptotene spermatocytes to traverse the BTB. During stages IX–XIV, SC junctions stabilize again, enabling progression through meiosis without compromising barrier integrity [19,20]. This stage-specific restructuring underscores the BTB’s adaptability, integrating junctional complexes and cytoskeletal dynamics to maintain immune privilege while accommodating germ cell migration.

### 2.1. Tight Junctions

Tight junctions (TJs) between adjacent SCs are critical components of the BTB, establishing basal-adluminal segregation necessary for proper germ cell development [12]. In epithelial cells, the apical junctional complex (AJC) includes TJs at the apical–lateral boundary and adherens junctions (AJs) just beneath. While each junction type possesses specific transmembrane and cytoplasmic proteins, they are functionally interconnected, coordinating barrier integrity and cellular signaling. During early embryogenesis, TJ and AJ proteins initially assemble as a shared complex, which later segregates to form distinct junctional structures characteristic of mature epithelia [21]. In the BTB, TJs function as a gate, restricting paracellular diffusion, and as a fence, preserving apico-basal polarity critical for compartmentalization, directional signaling, and germ cell maturation [3]. TJs are composed of three major groups of proteins that work cooperatively to maintain BTB function. Integral membrane proteins within TJs include five primary families: occludins, tricellulins, claudins, junctional adhesion molecules (JAMs), and Crumbs proteins [22,23]. Occludins, tricellulins, and claudins form the core sealing strands that regulate paracellular permeability, while tricellulin is highly enriched at sites where three epithelial cells meet. Similar to occludin, tricellulin interacts with the scaffolding protein zonula occludens-1 (ZO-1) through its cytoplasmic C-terminal domain, thereby establishing an indirect link with the F-actin cytoskeleton [24]. JAMs contribute to cell–cell adhesion and can influence immune cell migration [22], whereas Crumbs proteins are large transmembrane proteins that maintain Sertoli cell polarity [23]. Peripheral scaffolding proteins, including zonula occludens proteins, PDZ domain-containing proteins, and other signaling molecules, connect TJ components to the cytoskeleton and modulate intracellular signaling [25]. Among these, ZO-1 plays a central role in maintaining BTB integrity by linking claudin-11 and occludin to the actin cytoskeleton and stabilizing tight junctions. Experimental evidence shows that busulfan treatment in rats disrupts ZO-1 expression and localization in Sertoli cells, weakening barrier function and increasing BTB permeability [26]. Collectively, these findings highlight that, although traditionally viewed as static seals, TJs at the BTB are highly dynamic structures undergoing cyclical remodeling to facilitate germ cell movement during spermatogenesis.

### 2.2. Gap Junctions

In contrast to TJs, gap junctions (GJs) are not primary sealing elements of the BTB (unlike TJs), but rather intercellular channels that facilitate communication through up to a thousand connexon-based pores. A functional GJ channel consists of hemichannels (connexons) that dock between neighboring cells, each hemichannel being a group of six connexin protein subunits [5]. Through this structure, GJs permit the direct diffusion of small metabolites, ions, and second messengers, typically less than 1 kDa in size, between coupled cells [3]. Connexin 43 (Cx43) is the most significant GJ protein, essential for both the initiation and progression of spermatogenesis and for mediating communication between joined SCs. In addition, it regulates junction dynamics within the BTB. Cx43 expression levels correlate with spermatogenic status, and in cases of impaired spermatogenesis or male infertility, Cx43 is often dysregulated [27]. Given that connexins have an exceptionally short half-life (1–3 h), their synthesis must be a continuous and dynamic process to ensure ongoing replacement, which is needed for opening (gating), closing, assembly, and removal of Cx43 channels [28]. This is controlled by a variety of signaling pathways, most notably protein phosphorylation. Cx43 has a large number of serine and tyrosine units that are targets of several kinases that regulate gap junction remodeling.

### 2.3. Basal Ectoplasmic Specializations

The basal ectoplasmic specialization (ES), also known as zonula adherens, is a unique, testis-specific adherens junction between neighboring SCs [29]. It is made up of a densely packed actin filament network inserted between the plasma membrane and cisternae of the endoplasmic reticulum. The basal ES is crucial for the functional integrity and continuous restructuring needed during spermatogenesis. Being a structural specialization of the BTB, it provides strong cell–cell adhesion, often described as a “band” encircling the tubule near the basement membrane [30]. Through adhesion molecules, the basal ES not only anchors SCs together but also serves as a signaling center that integrates structural connections with intracellular regulatory pathways. This functional multifunctionality is supported by specific protein complexes that mediate both adhesion and signal transduction. The basal ES is an adhesion complex based on N-cadherin and β-catenin, with N-cadherin being linked to nonreceptor protein tyrosine kinases [6].

### 2.4. Desmosome-like Junctions

Desmosome-like junctions (DJs) represent a mechanically supportive yet dynamically regulated component of the BTB, where they coexist with TJs, basal Ess, and GJs to stabilize SC-SC adhesion. Structurally, integral membrane proteins such as desmogleins and desmocollins mediate adhesion between joined SCs, while their cytoplasmic domains are attached to the scaffolding network composed of intermediate filaments. A key cytoskeletal protein associated with these desmosome-like junction proteins is vimentin (Vim), which contributes to seminiferous epithelium stability and mechanical resilience [31].

Importantly, rather than being static structures, desmosome-like junctions are dynamically regulated. Their close molecular and signaling integration with SFKs and adaptor proteins enables crosstalk with neighboring junctional complexes. This supports DJs in participating in the controlled “opening” and “resealing” of the barrier during stage VIII spermatocyte transit [32,33]. As a result, perturbation of DJ composition or cytoskeletal anchoring is expected to disrupt BTB integrity, particularly under conditions that challenge junctional homeostasis.

## 3. BTB Remodeling Dynamics: Kinase Signaling and Cytoskeletal Interactions

Actin filaments are key components of the cytoskeleton that interact with focal adhesions, intercellular junctions, and the membrane cytoskeleton, playing a central role in maintaining barrier integrity in epithelia such as the BTB [34]. Together with polarized microtubules, actin filaments provide the flexibility required for dynamic remodeling, allowing developing germ cells to traverse the seminiferous epithelium efficiently. Actin filaments (F-actin) are polarized structures formed by the reversible polymerization of globular actin (G-actin) subunits, with a fast-growing barbed (plus) end and a slower pointed (minus) end, enabling rapid reorganization in response to cytoskeletal and signaling cues [35]. Filament elongation occurs primarily through the addition of ATP-bound G-actin monomers at the barbed end, followed by ATP hydrolysis, which drives filament turnover and continuous remodeling of the actin network [36]. At the ES, actin filaments are dynamically reorganized between bundled and branched configurations through the coordinated actions of actin-binding proteins (ABPs) [37].

### 3.1. Actin-Binding Proteins

ABPs regulate the dynamic turnover, stabilization, and higher-order organization of actin filaments at the BTB. Functionally, these proteins can be broadly classified into two categories: those that induce filament branching and those that facilitate the formation of bundled filament arrays [13]. Among these key regulator proteins, epidermal growth factor receptor substrate 8 (Eps8), a mediator of capping and bundling of barbed ends of filaments, and the Arp2/3 complex (composed of the assembly of Arp2, Arp3 and the ARPC 1–5 subunits), responsible for actin branching, emerge as key regulators in actin remodeling [38]. Together, these ABPs coordinate the structural plasticity required for SC junctional dynamics and germ cell translocation across the seminiferous epithelium.

Eps8 localizes to the actin-rich ES, where it functions either as an actin filament-bundling protein through its association with insulin receptor tyrosine kinase substrate p53 (IRSp53) or as a barbed-end capping protein when interacting with Abelson interacting protein-1 (Abi-1) [37]. Its barbed-end capping activity regulates filament length and limits the availability of free barbed ends required for actin branching [39]. In contrast, actin bundling organizes each actin filament into tightly packed parallel bundles, which is required for the ES to secure the attachment of developing spermatids to SCs [13].

On the other hand, Arp2/3 promotes the formation of a branched actin network composed of Y-shaped filaments by nucleating new filaments from the side of existing ones at a ~70° angle [40]. SCs express Neural Wiskott-Aldrich Syndrome Proteins (N-WASP) that promote this nucleation by interacting with Arp2/3 to catalyze the previously mentioned branched filament growth. This interaction then catalyzes the conversion of previously bundled actin fibers into an actin network [19]. This reorganization destabilizes both the basal and adluminal actin structures at the ES, thereby facilitating the translocation of preleptotene spermatocytes across the BTB [41]. The expression of key ABPs in focus at the ES is tightly stage-dependent during spermatogenesis. Eps8 is highly expressed at epithelial seminiferous stages V-VII; however, its level becomes almost undetectable at the onset of stage VIII [37]. In contrast, Arp3 reaches its highest levels during stage VIII, reflecting a switch from filament bundling to branching at the ES [42].

**Figure 1:**
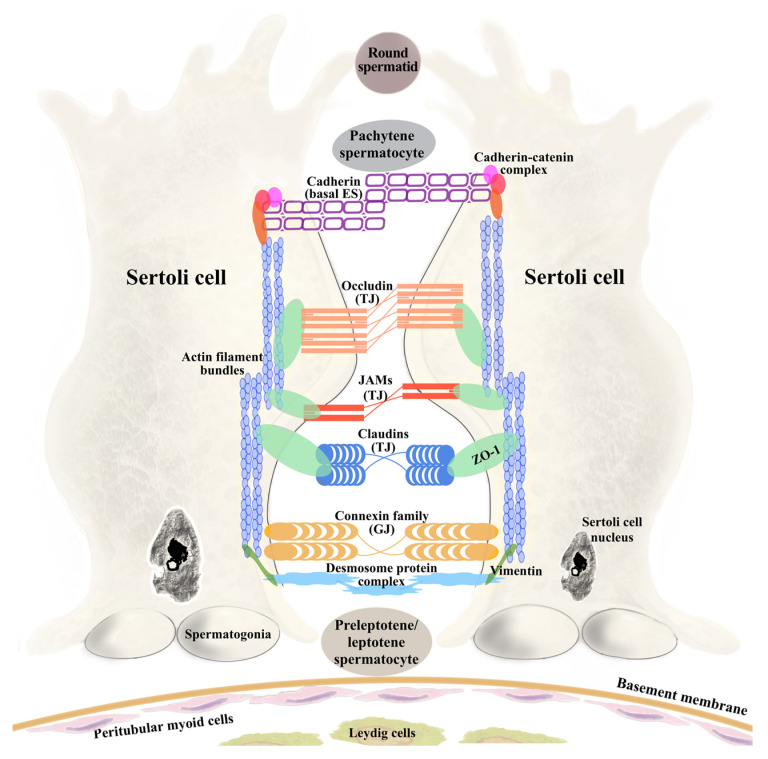
Schematic overview of the morphological features of the blood–testis barrier. ES: ectoplasmic specialization; GJ: gap junction; JAM: junctional adhesion molecules; TJ: tight junction; ZO-1: zonula occludens-1.

Palladin, an F-actin cross-linking protein, cooperates with Eps8 to stabilize actin filaments and maintain Sertoli–germ cell adhesion. Knockdown of palladin results in actin filaments being disorganized and unable to support TJ barrier function. Moreover, it leads to mislocalization of ZO-1, occludin and Eps8 in the palladin knockdown cells [43]. Palladin functions in coordination with Eps8, Arp2/3 and kinase-dependent signaling pathways to regulate the transition of actin filaments between bundled and branched configurations. At stage VII of the seminiferous epithelial cycle, phosphorylation-dependent modulation of palladin, associated with protein kinase activity, coincides with actin filament bundling and enrichment of Eps8 at the ES. During stage VIII, both palladin and Eps8 are displaced from the ES, allowing Arp2/3-mediated actin branching, which facilitates the translocation of preleptotene spermatocytes across the BTB [41]. Figure 1 shows details of BTB components between Sertoli cells and the most common junctional proteins and actin cytoskeletal organization in the testis.

### 3.2. Kinase Signaling

As discussed previously, a defining feature of the BTB is the presence of actin filament bundles and microtubules between SCs, which facilitate the internalization and remodeling of intact junctional complexes through protein kinase–regulated cytoskeletal dynamics [44]. Protein kinases (PKs) modulate protein function by catalyzing the transfer of phosphate groups from adenosine triphosphate (ATP) to serine, threonine, and/or tyrosine residues, which represent the predominant sites of cellular phosphorylation. Through these phosphorylation-dependent mechanisms, PKs regulate the assembly and disassembly of BTB junctions [45]. Dysregulation of these signaling pathways impairs normal barrier dynamics and leads to increased BTB permeability, ultimately compromising spermatogenic function [46]. Beyond kinase families directly associated with junctional remodeling, accumulating evidence indicates that major inflammatory signaling pathways—particularly NF-κB-dependent signaling, as well as JAK/STAT and MAPK cascades—are activated in multiple rodent models relevant to BTB stress and inflammation. In Sertoli cells, NF-κB signaling has been shown to regulate TJ protein dynamics, as demonstrated in Sertoli cell culture systems where NF-κB-dependent alterations in occludin and ZO-1 expression were observed [47]. Similarly, JAK/STAT signaling contributes to BTB disruption, with elevated JAK2/STAT3 activity being associated with reduced expression of key junctional proteins, including claudin-11, connexin-43, and ZO-1, whereas pharmacological inhibition of JAK2 preserves junctional integrity [48]. In addition, inflammatory cytokines such as interleukin-6 (IL-6) activate MAPK cascades, including ERK1/2, in Sertoli cells, leading to altered TJ protein dynamics and impaired BTB function [49]. These “master” inflammatory pathways do not act in isolation but converge on cytoskeletal regulators and junction-associated proteins that ultimately determine BTB stability.

This review focuses on the interplay between inflammatory signaling, cytoskeletal dynamics, and junctional regulation at the BTB, with Src family kinases serving as one example of kinases integrating extracellular signals with barrier remodeling. Src family kinases (SFKs) generally consist of six distinct domains, although domain composition and functional contributions may vary among family members. Together, these domains regulate their cellular localization, interactions, and enzymatic activity. The Src homology 1 (SH1) domain forms the catalytic core of SFKs and is responsible for their tyrosine kinase activity, which is tightly controlled by phosphorylation events that toggle the kinase between active and inactive states. A negative regulatory tail at the C-terminus maintains the kinase in an autoinhibited conformation [50]. SFKs have emerged as key regulators in the BTB remodeling dynamics, as several members of this family, including Fyn, c-Yes, and c-Src, are highly expressed at ES junctions. These kinases form functional complexes with ABPs; for example, Fyn associates with both Arp3 and Eps8 in the testis, thereby directing either junction disassembly or stabilization depending on the stage of the seminiferous epithelial cycle [30].

#### 3.2.1. c-Yes

Protein kinase c-Yes, similar to other Src family members, comprises several distinct functional domains. The SH4 domain mediates protein attachment to cell membranes, while the SH3 and SH2 domains facilitate c-Yes interaction with other proteins, and the SH1 domain is responsible for the enzymatic activity of the protein kinase [50]. Notably, among the ABPs, c-Yes has been shown to physically interact with Eps8, but not with Arp3 [51]. This selective interaction highlights that, in cooperation with palladin and Eps8, c-Yes contributes to actin filament bundling (rather than remodeling) and stabilization, thereby reinforcing SC junctions and maintaining BTB integrity [41]. Furthermore, c-Yes physically associates with several key components of junctional complexes. It structurally interacts with occludin, but it does not interact with other TJ proteins like CAR or JAM-A. At the basal ES, it associates with the integral membrane protein N-cadherin and the adaptor protein β-catenin [52]. Together, these selective interactions position c-Yes as a stabilizing Src family kinase that reinforces established junctional architecture at the BTB.

#### 3.2.2. c-Src

In contrast to the selective, stabilizing interactions mediated by c-Yes, c-Src targets a broader spectrum of junctional complexes, in line with its role in BTB restructuring. Similarly to c-Yes, c-Src is composed of multiple structural elements that regulate its activity. From the N-terminus, the SH4, SH3, and SH2 domains are highly conserved and play key regulatory roles, followed by the SH1 catalytic domain. Notably, the SH2 and SH3 domains modulate kinase activity by controlling the function of the SH1 catalytic domain through intramolecular interactions that control the regulation of c-Src signaling [53]. Functionally, c-Src appears to exert distinct effects on BTB remodeling, as it has been implicated in regulating the nucleation activity of Arp3 [54]. Within the BTB, SFKs coordinate whether the actin cytoskeleton adopts a bundled or branched configuration through their ability to directly activate N-WASP—leading to Arp2/3 complex activation—and by modulating Eps8 function via tyrosine phosphorylation [55]. Consistent with its role in coordinating actin remodeling at the basal ES, c-Src localizes to junctional protein complexes, thereby linking cytoskeletal regulation to Sertoli–Sertoli cell adhesion. Supporting this notion, immunoprecipitation analyses of adult rat seminiferous tubule extracts demonstrated that c-Src is physically associated with the adhesion proteins N-cadherin and β-catenin within the same protein complex [56].

Furthermore, knockdown of desmoglein-2 and desmocollin-2 disrupted c-Src localization and resulted in impaired barrier integrity, supporting a functional link between c-Src and DJs at the BTB [57]. Consistent with its function as a junction-remodeling kinase, c-Src also regulates other Sertoli–Sertoli junctional complexes at the BTB, including GJs. Specifically, Src activation inhibits gap junctional intercellular communication (GJIC) by targeting tyrosine residues on connexin-43 (Cx43), promoting connexosome formation and subsequent gap junction disassembly [5].

#### 3.2.3. Fyn

**Figure 2:**
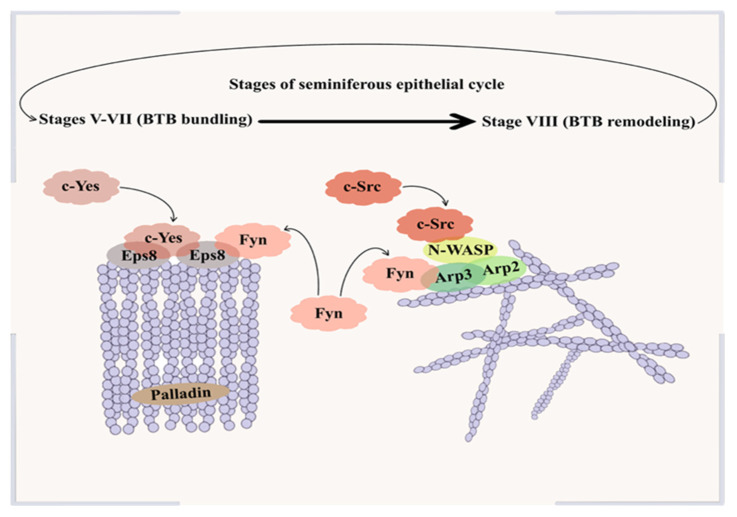
Stage-specific roles of Src family kinases in actin-based BTB remodeling during the seminiferous epithelial cycles in rodents.

Fyn is an Src family kinase that shares the conserved structural framework characteristic of other family members. Its N-terminal region mediates plasma membrane binding, while, similar to c-Src and c-Yes, the SH2 and SH3 domains facilitate protein–protein interactions and contribute to the regulation of kinase activity. The SH1 domain contains the ATP-binding site and serves as the catalytic function regulated by phosphorylation-dependent conformational changes [58]. Fyn physically associates with ABPs, including Arp3 and Eps8, thereby influencing the balance between bundled and branched actin filament configurations. Increased interaction of Fyn with Arp3 promotes actin branching and junctional remodeling, increasing BTB permeability, whereas its interaction with Eps8 supports actin bundling and junctional stability. This dual regulatory capacity identifies Fyn as a key modulator of BTB restructuring under both physiological and stress-induced conditions [30]. Figure 2 shows the role of c-Yes, c-Src, and Fyn in the regulation of BTB remodeling during the stages of epithelial cycles.

## 4. Inflammation and BTB Permeability

Inflammation represents a major physiological and pathological challenge to the integrity and function of the BTB. Inflammatory conditions within the testis—arising from both exogenous and endogenous factors, including infection, environmental effects, oxidative stress, and adverse endocrine changes—are closely associated with impaired spermatogenesis and reduced sperm quality [59,60,61,62]. Accumulating evidence indicates that inflammatory responses actively interfere with the molecular mechanisms that govern BTB dynamics. Proinflammatory cytokines, chemokines, reactive oxygen species, and lipid mediators can increase BTB permeability and compromise immune privilege [63,64]. Notably, inflammation-induced BTB alterations frequently precede overt germ cell loss, supporting the concept that barrier disruption constitutes an early and causative event in inflammation-associated testicular pathology.

At the cellular level, inflammatory signaling intersects with key regulatory pathways that control BTB remodeling. ABPs, junctional complexes, and kinase-associated signaling—including SFKs—are particularly sensitive to inflammatory cues [30]. Through these pathways, inflammation can shift the equilibrium from a stabilized, bundled cytoskeletal architecture toward a more disorganized, permissive state, thereby rendering the BTB more vulnerable to disruption [65,66,67]. Accumulating evidence suggests that acute and chronic inflammatory signaling exert fundamentally distinct effects on BTB dynamics [68]. Acute inflammatory models, most commonly involving intratesticular administration of pro-inflammatory cytokines such as TNF-α, IL-1, or TGF-β3, induce rapid but largely reversible BTB remodeling. These changes are characterized by transient relocalization of junctional proteins, reorganization of the actin cytoskeleton, and a controlled increase in barrier permeability that facilitates germ cell transit without causing permanent structural damage [69,70]. In contrast, chronic inflammatory conditions—including autoimmune orchitis and chemotherapy-induced testicular injury—are associated with sustained cytokine exposure, oxidative stress, and persistent activation of inflammatory signaling pathways. Under these conditions, BTB disruption is no longer adaptive but becomes pathological, marked by reduced expression of junctional proteins, collapse of cytoskeletal architecture, and prolonged loss of barrier integrity. Sertoli cell stress and impaired cytoskeletal support further compromise the stability of the spermatogenic niche, ultimately leading to defective spermatogenesis and infertility [71,72,73].

Importantly, emerging evidence from human studies supports the clinical relevance of inflammation-induced BTB dysfunction described in experimental models. Although notable differences exist between human and rodent reproductive physiology—such as variations in the duration and organization of spermatogenic stages—which may limit direct translational extrapolation, multiple clinical observations indicate that inflammatory conditions in the human testis are associated with molecular and structural alterations of the BTB. Specifically, BTB damage has been associated with reduced expression or mislocalization of junctional proteins—including ZO-1, ZO-2, connexin-43, and claudin-11—in patients with testicular carcinoma in situ [74], type 2 diabetes mellitus [71], and conditions such as maturation arrest at the level of primary spermatocytes or Sertoli cell-only syndrome [75]. Furthermore, testicular inflammation associated with viral infections, such as COVID-19, correlates with decreased expression of these BTB components and a reduced number of Sertoli cells [76].

In addition to mechanistic insights, several pharmacological strategies have been shown to mitigate inflammation-induced BTB disruption in experimental models. Polyphenolic compounds (e.g., curcumin, quercetin, luteolin, and resveratrol) [77,78,79,80] and classical antioxidants, such as vitamins C and E, and melatonin [81,82], have been reported to preserve tight junction organization and limit oxidative stress-driven BTB dysfunction in rodent and Sertoli cell models. Beyond antioxidant approaches, pathway-specific modulators (SB431542, SB203580, pomalidomide) [83,84,85], as well as atorvastatin and ML221, demonstrate protective effects on BTB structure and barrier properties, with evidence also observed in human Sertoli cell culture systems [71,86]. Despite these promising preclinical findings, challenges related to bioavailability, specificity, and long-term safety currently limit direct clinical translation.

Collectively, these findings suggest that inflammation-driven disruption of BTB components observed in experimental systems reflects clinically relevant mechanisms contributing to human male infertility. Thus, rodent models focusing on inflammatory regulation of BTB dynamics provide a valuable framework for dissecting the molecular pathways underlying disease-associated spermatogenic failure.

Accordingly, the subsequent sections distinguish between experimental models based on direct cytokine exposure and disease-associated inflammatory conditions, including those characterized by reproductive dysfunction accompanied by compromised BTB integrity.

### 4.1. Effects of Exogenously Administered Pro-Inflammatory Cytokines on BTB Elements and Dynamics in Experimental Models

#### 4.1.1. Interleukin-6 (IL-6)

Under inflammatory conditions, cytokines can shift the tightly regulated dynamics of BTB toward a pathological “leaky” state, primarily by altering TJ protein levels, localization, and the balance between endocytosis and recycling. This phenomenon was supported by Zhang et al., who provided mechanistic insight into the roles of IL-6 in regulating BTB dynamics. The in vitro results showed that IL-6 alters the localization and amount of BTB-constituent proteins, while their mRNA levels remain unchanged. The turnover of occludin, JAM-A, and N-cadherin is delayed, leading to their accumulation in SCs, which compromises TJ organization, increases barrier permeability, and underlies BTB dysfunction [49].

#### 4.1.2. Tumor Necrosis Factor-Alpha (TNF-α) and Interleukin-17 (IL-17)

Extending the discussion on cytokine-mediated BTB regulation, TNF-α has also been shown to induce changes in tight junction protein localization and barrier integrity. In a previous in vivo study, Li et al. demonstrated that local administration of TNF-α to adult rat testes transiently disrupted the BTB, reducing the protein levels of occludin, ZO-1, and N-cadherin, increasing barrier permeability, and transiently activating p38 and ERK MAPK signaling, suggesting that localized TNF-α production by Sertoli and germ cells facilitates the timely restructuring of the BTB to allow spermatocyte transit [69]. Similar to TNF-α-induced alterations, IL-17 impaired SC barrier integrity both in vitro and in vivo by reducing the expression of occludin and altering claudin 11 distribution. Intratesticular injection of IL-17 induced recruitment of immune cells and resulted in germ cell sloughing and apoptosis [87].

Together, these findings indicate that cytokine-induced BTB disruption extends to additional adhesion complexes that also regulate barrier stability. In this context, DJs emerge as secondary targets of inflammatory signaling, linking cytokine exposure to impaired cytoskeletal remodeling and mechanical adhesion, given that within the BTB, DJs represent an additional adhesive element that supports Sertoli-Sertoli junctional integrity during active barrier remodeling. Although these junctions are structurally robust under physiological conditions, accumulating evidence indicates that inflammatory stimuli promote junctional destabilization. These hybrid junctions are built around desmosomal cadherins, primarily desmoglein-2 (DSG2) and desmocollin-2 (DSC2), which are anchored to intermediate filaments, such as Vim, and associate with adaptor and signaling proteins, enabling both mechanical adhesion and signaling-dependent barrier regulation [31,65]. Controlled in vitro systems have been essential for defining the mechanisms by which inflammatory cytokines impair BTB junctions. Lydka et al. demonstrated in a primary SC culture model that exposure to TNF-α induces significant alterations in cell–cell adhesion complexes, accompanied by reorganization of the actin cytoskeleton, causing destabilization of junctional architecture under inflammatory conditions [88]. Supporting these findings, Noguchi et al. suggested, using an inflammation-associated SC dysfunction model, that pro-inflammatory cytokine exposure leads to loss of DSG2 from DJs and disruption of the vimentin cytoskeleton, thereby contributing to BTB dysfunction and impaired spermatogenesis [89]. Importantly, these inflammatory effects occur at epithelial stages when the BTB is already undergoing active cytoskeletal reorganization. Another model by Li et al. showed that exogenously administered TNF-α induced inflammatory signaling that disrupts BTB integrity through activation of the p38 MAP kinase signaling pathway in a stage-dependent manner, consistent with seminiferous epithelial cycle stage (late) VII and stage VIII, where the well-described transition from bundled to branched F-actin organizations is regulated by ABPs such as Eps8 and the Arp2/3 complex. This overlap supports the possibility that inflammatory signaling may exploit these stage-dependent cytoskeletal dynamics [69].

#### 4.1.3. Transforming Growth Factor-Beta (TGF-β)

Members of the transforming growth factor-beta (TGF-β) family are expressed by Sertoli and immune cells located in the testicular interstitium and constitute important inflammatory regulators of BTB function. During in vitro assembly of inter-Sertoli TJs, TGF-β3 dose-dependently disrupts the BTB by inhibiting the transient and basal expression of key TJ proteins, including occludin, ZO-1, and claudin-11. These findings indicate that TGF-β3 plays a critical role in modulating the molecular events underlying SC junction assembly [90]. Using a primary Sertoli cell culture model, Wong et al. demonstrated that TGF-β3 accelerates the endocytosis of integral junction proteins without immediately affecting their steady-state levels. Furthermore, it has been shown that Cdc42, a Rho family small GTPase actin cytoskeleton regulator, is a crucial component in the TGF-β3–mediated cascade, which can lead to the disruption of the TJ fibrils [91]. A similar mechanism occurs at the basal ES with TGF-β2, which destabilizes basal ES architecture by promoting N-cadherin endocytosis and directing internalized proteins toward late endosomal degradation rather than recycling to the Sertoli cell surface, thereby rapidly reducing functional adhesion complexes [92].

#### 4.1.4. Interleukin-1-Alpha (IL-1α)

In a previous study, Sarkar et al. aimed to examine the effects of IL-1α on spermatogenesis. Their findings showed that intratesticular administration of IL-1α profoundly compromises Sertoli–germ cell adhesion and BTB integrity in a stage-dependent manner. Early sloughing of elongated spermatids at stages VII–VIII precedes widespread disruption of germ cell adhesion. Importantly, IL-1α does not alter the steady-state expression levels of key BTB constituent proteins, including occludin, claudin-1, JAM-A, ZO-1, and N-cadherin; instead, it induces their mislocalization away from sites of cell–cell contact. Concomitantly, IL-1α disrupts the highly ordered organization of filamentous actin at both the BTB and the apical ectoplasmic specialization, with focal loss of actin filaments and collapse of the Sertoli cell cytoskeleton, which can eventually lead to spermatogenic arrest. Lie et al. further support this mechanism using an in vitro Sertoli cell model. Their findings suggest that IL-1α-induced BTB disruption preferentially targets molecular contexts resembling stage VIII of the seminiferous epithelial cycle, when Eps8 expression declines and Arp3 levels are elevated [93].

Extending these observations to other junctional components of the BTB, inflammatory conditions also compromise GJ. Chojnacka et al. demonstrated in a primary Sertoli cell culture model that exposure to the proinflammatory cytokine IL-1α increased total Cx43 protein levels together with its inhibitory phosphorylated form at Ser368. Despite this apparent upregulation, GJIC was markedly reduced, indicating that inflammatory signaling functionally uncouples Cx43 at the BTB. This impairment was accompanied by altered organization of BTB-associated structural elements, including changes in ZO-1 localization and pronounced mislocalization of F-actin and palladin, resulting in cytoskeletal disorganization. Moreover, inflammatory exposure reduced the actin-bundling capacity of Sertoli cell lysates and promoted peripheral actin aggregation, thereby destabilizing the actin anchoring required for effective Cx43-mediated intercellular communication [7]. To further support this concept, another in vitro study by Mickus et al. demonstrated that kinase-dependent phosphorylation of Cx43 is a key regulator of GJ functionality. Notably, phosphorylation at Ser368 by protein kinase C (PKC) suppresses GJIC by promoting channel closure and destabilization of junctional plaques [8].

### 4.2. Impact of Inflammation on BTB Elements and Dynamics in Experimental Models of Reproductive Dysfunction Associated with BTB Disruption

Disease models that mimic sustained inflammation and testicular disruption provide valuable insight into how chronic inflammatory conditions compromise BTB integrity in pathological conditions. The BTB represents a major target of environmental toxicants and endocrine-disrupting chemicals, including heavy metals and pesticides widely used in industrial, agricultural, and domestic settings. Among them, cadmium has been shown to significantly reduce the levels of adhesion proteins, including ZO-1, occludin, and E-cadherin [94,95]. In addition, exposure to organophosphate pesticides and microplastics has been associated with increased BTB permeability [96,97]. Beyond these endocrine-disrupting chemicals, it is important to highlight the role of pathological conditions and diseases, which influence reproductive function through inflammatory and oxidative mechanisms acting on the BTB. Diabetes mellitus represents a prominent example, as accumulating evidence suggests that chronic metabolic and inflammatory stress in diabetes is associated with BTB dysfunction and altered Sertoli cell junctional organization. Wei et al. reported that hyperglycemia and a consequent oxidative stress resulted in a reduced expression and function of ZO-1 and Cx43 [98].

Using a model of experimental autoimmune orchitis characterized by extensive seminiferous epithelial disruption, Pérez et al. analyzed alterations in BTB permeability and TJ protein dynamics. Their findings clearly show that early germ cell sloughing coincides with reduced occludin expression, mislocalization of claudin-11 and ZO-1, and increased BTB permeability in the experimental orchitis rat model. Moreover, IL-6 contributed to these alterations, as it induces seminiferous tubule damage in vivo and causes TJ protein redistribution and reduced barrier function in cultured Sertoli cells [73]. In addition to the autoimmune orchitis model, the decline in the expression of TJ proteins and a consequent disruption of the BTB have been underpinned in further experimental models, such as cryptorchidism [99] and spinal cord injury [100].

In addition to cytokine-driven inflammatory conditions, it is also essential to consider disease models of reproductive dysfunction in which BTB impairment arises as an adverse consequence of anticancer chemotherapy. He et al. used a fluoride-induced inflammation model to investigate the vulnerability of ABP-regulated cytoskeletal architecture to inflammatory stress. In this in vivo system, animals were treated with fluoride for 30 days, which resulted in elevated intratesticular levels of IL-1α. These changes coincided with a significant increase in Arp3 levels, associated with a reduced F-actin bundling and a marked shift toward a branched F-actin configuration [101]. In a recent study, Zhao et al. examined the effects of intratesticular busulfan injection on BTB integrity and the spermatogenic microenvironment within a 21-day-long period. Busulfan, a chemotherapeutic alkylating agent, induces pronounced testicular inflammation, characterized by interstitial immune cell infiltration, vacuolation of the seminiferous tubule wall, and progressive germ cell sloughing. In parallel, levels of inflammatory cytokines, including TNF-α, IL-1β, and IL-6, rise above physiological limits, correlating with disrupted BTB integrity. Key BTB proteins (i.e., occludin, N-cadherin, and Cx43), along with supporting cytoskeletal elements such as vimentin, gradually decrease, accompanied by impaired continuity of the BTB structure and altered androgen receptor expression [102]. Similar to busulfan, cyclophosphamide—an alkylating agent—has been shown to elevate pro-inflammatory cytokine levels and activate signaling pathways that compromise testicular immune privilege in rodent models. In a recent study, Cellat et al. demonstrated that a 56-day cyclophosphamide treatment triggered a pronounced testicular inflammatory cascade characterized by NF-κB activation and increased TNF-α and IL-6 levels, accompanied by inflammation-driven impairment of Sertoli cell adhesion and barrier integrity [103]. Consistent with these findings, cyclophosphamide-induced testicular damage has also been reported in other experimental models, where reduced occludin expression and elevated TGF-β3 levels were observed following treatment [104].

There are earlier studies implicating Fyn, c-Yes, and c-Src kinases in BTB regulation and junctional dynamics; however, direct evidence linking these kinases specifically to inflammatory cytokine signaling at the BTB is still emerging. Nevertheless, the existing literature clearly demonstrates that Src family kinases play critical roles in BTB architecture, cytoskeletal organization, and junctional protein trafficking. Given their central involvement in maintaining BTB integrity and coordinating dynamic junction remodeling, the lack of mechanistic studies directly addressing Src kinase-mediated inflammatory signaling at the BTB represents a critical gap in the field and underscores the need for further experimental studies. Table 1 summarizes the rodent models of inflammation-associated BTB disruption.

## 5. Conclusions

The BTB represents a uniquely dynamic system that preserves immune privilege while undergoing tightly regulated, stage-specific remodeling to support spermatogenesis. The evidence reviewed here collectively demonstrates that inflammatory conditions disrupt this delicate balance, with both acute and chronic inflammatory signals exerting distinct effects on BTB structure and function. While intratesticular cytokine-based models have provided valuable mechanistic insight into cytokine-specific regulation of junctional proteins, disease-associated models such as orchitis and chemotherapeutic injury underscore the pathological consequences of sustained inflammatory signaling and persistent barrier impairment.

Future studies should aim to delineate how inflammatory pathways temporally and spatially converge on cytoskeletal regulators and kinase signaling networks at the BTB. Integrating advanced approaches—including single-cell and spatial profiling and refined in vivo models that better recapitulate chronic inflammatory states—seems to be essential to bridge current translational gaps. Such efforts will be critical for guiding the rational development of strategies to preserve male reproductive function under inflammatory conditions.

## Figures and Tables

**Table 1:** Experimental models of inflammation-associated BTB disruption.

Induction of Inflammation	Affected Proteins	Mechanism of Action	Refs.
**Exogenous Cytokine-Mediated Effects on BTB Components in Experimental Models**			
IL-6	Occludin, JAM-a, N-cadherin, β-catenin, Claudin-11	Delayed occludin, JAM-a & N-cadherin degradation + occludin & β-catenin mislocalization → disrupted TJ barrier → impaired BTB permeability	[49,73]
		↑ CD45+ level → ↓ Occludin expression & claudin 11 mislocalization → altered TJ structure and function → disrupted BTB integrity	
IL-17	Occludin, Claudin-11	↑ CCL2 expression & ED1+ macrophage infiltration → occludin & claudin 11 redistribution + ↓ occluding expression → altered TJ barrier function and increased BTB permeability	[87]
IL-1α	F-actin, Occludin, EPS8, Arp3, ZO-1, N-cadherin, β-catenin	Uneven distribution of F-actin, EPS8 delocalization (cell–cell interface → nucleus) & ↑ Arp3 expression + delayed degradation & mislocalization of occludin, ZO-1, N-cadherin and β-catenin	[7,8,93]
		↑ Connexin-43 level & phosphorylation + peripheric F-actin, cortactin & palladin aggregation → ↓ GJ functionality & BTB dysfunction	
TNF-α	Occludin, ZO-1, N-chaderin, F-actin, Claudin-11, Connexin-43, Desmoglein-2	↑ ERK & p38 MAPK signaling → ↓ occludin, ZO-1 & N-cadherin levels + defragmentation & peripheric aggregation of F-actin → disrupted BTB function & increased permeability	[69,88,89]
		↓ Connexin 43 & desmoglein 2 expression + claudin 11 & ZO-1 mislocalization + aberrant F-actin & vimentin distribution → germ cell exfoliation and BTB dysfunction	
TGF-β3	Occludin, ZO-1, Claudin-11, CAR	↓ Occludin & Claudin-11 (suppressed induction) + ↓ ZO-1 expression → impaired TJ assembly → BTB weakening	[90,91]
		↑ TGF-ß3 → ZO-1 mislocalization (surface → cytosol) → enhanced endocytosis → BTB destabilization	
TGF-β2	Occludin, N-cadherin	↑ Occludin & N-cadherin endocytosis → BTB destabilization	[92]
**Experimental models of reproductive dysfunction associated with BTB disruption**			
Cryptorchidism	Claudin-11, Connexin-43	Chronic inflammation → ↓ Claudin-11 & Connexin-43 expression → Sertoli cell dysfunction → BTB disruption	[99]
Spinal cord injury	Occludin	Testicular inflammation (↑ IL-1ß, immune-cell infiltration) → ↓ Occludin expression & mislocalization → BTB disruption	[100]
Orchitis	Occludin, Claudin-11, ZO-1	Testicular inflammation (& ↑ IL-6) → ↓ Occludin expression + Claudin-11 & ZO-1 delocalization → ↑ BTB permeability → BTB disruption and germ cell sloughing	[73]
Fluoride	Arp3, F-actin, ß-catenin	(↑ IL-1α) → ↑ Arp3 expression → highly branching F-actin & ↓ F-actin expression → ↓ basal ES (↓ β-catenin expression) → BTB functional & ultrastructural disruption	[101]
Busulfan	N-cadherin, Occludin, Connexin-43, Vimentin	Testicular inflammation (↑ TNF-α, IL-6, IL-1ß) → ↓ Occludin, Connexin-43, N-cadherin, Vimentin content decreased and discontinuous distribution → BTB structural disruption	[102]
Cyclophosphamide	Occludin	Oxidative stress & Testicular inflammation (NF-κB → ↑ TNF-α & IL-6) → reproductive toxicity Testicular toxicity (↑ TGF-ß3) → ↓ Occludin expression and germ cell loss → BTB-related testicular injury	[103,104]

↑: increased; ↓: decreased.

## Data Availability

No new data were created or analyzed in this study.

## Abbreviations

The following abbreviations are used in this manuscript:

ABPs	actin-binding proteins
BTB	blood–testis barrier
DS	desmosome-like junction
ES	ectoplasmic specialization
GJs	gap junctions
SC	Sertoli cell
SFK	Src family kinase
TJs	tight junctions
